# Rituximab for the treatment of idiopathic membranous nephropathy with nephrotic syndrome: a systematic review and meta-analysis

**DOI:** 10.3906/sag-2104-177

**Published:** 2021-08-14

**Authors:** Lu YOU, Peiyi YE, Guanqing XIAO, Jiabao LIANG, Yaozhong KONG

**Affiliations:** 1 Department of Nephrology, The First People’s Hospital of Foshan, Foshan, Guangdong, China; 2 Department of Hematology, Affiliated Nanhai Hospital of Southern Medical University, Foshan, Guangdong, China

**Keywords:** Rituximab, membranous nephropathy, immunosuppressive treatment, meta-analysis

## Abstract

**Background/aim:**

This meta-analysis comprehensively investigated the efficacy and safety of rituximab (RTX) in patients with idiopathic membranous nephropathy (IMN).

**Materials and methods:**

We searched the MEDLINE, EMBASE and Cochrane Registry of Controlled Trials databases from January 2000 to January 2020. Studies evaluating the efficacy and safety of RTX in the treatment of IMN with nephrotic syndrome (NS) were included.

**Results:**

Nine studies (total of 357 patients) were included in the meta-analysis. The pooled complete response and overall response (OR) rates at 12 months were 13.2% [95% confidence interval (CI), 0.09–0.18] and 60% (95% CI, 0.48–0.72), and those at 24 months were 27.8% (95% CI, 0.22–0.34) and 66% (95% CI, 0.6–0.72), respectively. The pooled OR rates for the low-, standard-, and high-dose groups were 39.3%, 64%, and 60%, respectively, and those for the first-line and second-line groups were 58% and 54%, respectively.

**Conclusion:**

Treatment of IMN with RTX has comparable efficacy to other immunosuppressive treatments (ISTs). RTX has the advantages of no requirement for steroids and lower rates adverse event and relapse rates. Patients who relapse or are resistant to other IST agents also respond to RTX. RTX-based regimens and other B-cell-targeted therapies may represent the future of IMN therapy.

## 1. Introduction

Membranous nephropathy (MN) is an antibody-mediated autoimmune glomerular disease characterized by membrane-like thickening of the glomerular basement membrane, caused by subepithelial immune-complex deposition on the outer aspect of the membrane. In total, 80% of MN cases are kidney-specific (idiopathic membranous nephropathy, IMN) and 20% are associated with other systemic diseases or exposures (secondary MN) [1]. IMN remains the leading cause of adult nephrotic syndrome (NS). About 20%–30% of IMN patients show spontaneous remission, while 30%–50% of those who progress to NS will experience end-stage renal disease (ESRD) within 5–10 years [2]. About 70%–80% of patients with IMN have circulating autoantibodies to the M-type phospholipase A2 receptor (PLA2R), which is expressed on podocytes, and 3%–5% have circulating antibodies to thrombospondin type-1 domain-containing 7A (THSD7A) [3,4]. In the remaining patients, the target antigen remains unidentified.

Recognition that IMN is an autoimmune disease has dramatically altered both the diagnostic and therapeutic approach. Current therapeutic guidelines recommend first-line IST with a modified Ponticelli regimen (6 months of alternating cycles of steroids and cyclophosphamide) for patients with proteinuria that do not respond to supportive care after 6 months, and for those with compromised baseline renal function [5,6]. This protocol leads to remission of proteinuria in about 50%–60% of patients within 12 months, and 70%–80% within 24–36 months, and is also associated with a low relapse rate and reduction in the rate of subsequent ESRD from 30%–40% to ≤ 10% [5,6]. Although the Ponticelli regimen and other similar alkylating agents and steroids have well-established efficacy, they also have relatively high rates of adverse events, including myelosuppression, infection, infertility, and later malignancy. The calcineurin inhibitors (CNIs) cyclosporine and tacrolimus, either used as monotherapies or combined with low-dose steroids, have been shown to decrease proteinuria and the rate of loss of renal function in IMN [7]. Because CNIs have lower incidence rates of infection and malignancy compared with alkylating agents, and are also effective as monotherapies, many clinicians prefer to initiate therapy with CNIs to avoid the more severe adverse events (SAEs) associated with cytotoxic agents and higher-dose steroids. However, long-term nephrotoxicity, the need to closely monitor drug levels, and the higher relapse rates associated with CNIs are considerable concerns [8]. The latest Kidney Disease Improving Global Outcomes (KDIGO) guidelines restricted the indication for alkylating agents to patients at high risk of progression, and consider CNIs as an alternative therapy [6].

Rituximab (RTX), a monoclonal antibody against the CD20 antigen present in B lymphocytes, was approved by the US Food and Drug Administration for the treatment of non-Hodgkin’ s lymphoma in 1997 [9]. Because the CD20 antigen is not expressed in hematopoietic stem cells and other normal tissues, selective B-cell depletion by RTX inhibits the production of autoantibodies involved in the pathogenesis of IMN, without the toxicity associated with nonspecific immunosuppression or any risk of secondary cancer [9]. Recent studies have revealed that RTX treatment of IMN has comparable outcomes to immunosuppressive alkylating-agent-based regimens [10]. Thus, RTX treatment may be an alternative to the alkylating-agent-based regimens or CNIs recommended by KDIGO as first-line treatments for IMN with NS. RTX-based regimens and other B-cell-targeted therapies may represent the future of IMN therapy. However, variable efficacy and a short track record of use, together with few published randomized controlled trials (RCTs), have resulted in inconsistent conclusions regarding RTX. It remains unknown whether RTX is equally effective in patients who failed to respond to previous IST, or what the most appropriate RTX dose or protocol is for the treatment of IMN. This meta-analysis aimed to comprehensively investigate the efficacy and safety of RTX in patients with IMN.

## 2. Materials and methods

### 2.1. Search strategy and inclusion criteria

We searched the MEDLINE, EMBASE, and Cochrane Registry of Controlled Trials databases from January 2000 to January 2020. We also searched the references of all identified studies, as well as related review papers. We used the following search terms: (primary OR idiopathic) AND (membranous nephropathy OR membranous glomerulonephritis) AND (rituximab OR anti-CD20 monoclonal antibody) AND nephrotic syndrome. Studies evaluating the efficacy and safety of RTX for the treatment of IMN with NS were included. Articles on secondary MN, other pathological types of glomerular diseases, and/or disease recurrence after renal transplantation, and those that were not full text or had a sample size of < 10 patients were excluded. Studies evaluating the outcomes of RTX combined with other IST drugs were also excluded. Two reviewers (LY and PYY) screened the titles and abstracts of all identified studies, to evaluate their eligibility for inclusion.

### 2.2. Data extraction and quality assessment

Three researchers (GQX, JBL, and LY) respectively extracted the following data for each study: first author, study region, publication year, study design, patient baseline characteristics, RTX dose, follow-up time, and study outcomes. We extracted data only from the RTX arm of RCTs, or the parts of studies that met the selection criteria. Disagreements among the three reviewers were resolved via discussion. Two colleagues (PYY and JBL) evaluated the quality of the included studies using the Newcastle–Ottawa Scale (NOS) [11], which is composed of eight items classified into three dimensions: selection (four items), comparability (one item), and exposure (three items). A maximum of one star can be awarded to a study for each item within the selection and exposure categories, and a maximum of two stars can be given for comparability. The studies were divided into three quality categories: low quality (scores 1–4), intermediate quality (scores 5–7), and high quality (scores 8–10).

### 2.3. Definition of outcomes

The primary outcomes of this study were the complete response (CR) rate, partial response (PR) rate, overall response (OR) rate, and relapse rate. Secondary endpoints were laboratory outcomes, including serum albumin and serum triglycerides, cholesterol, changes of renal function, CD19/CD20-positive B-cell counts, and anti-PLA2R depletion, as well as adverse events. CR was defined as a proteinuria level of no more than 0.5 g/day; PR was defined as a reduction in proteinuria of at least 50% from baseline, plus a final proteinuria level of 0.5–3.5 g/day; and OR was defined as CR+PR. No response was defined as the lack of a reduction of at least 25% in proteinuria from baseline. Relapse was defined as a proteinuria level of more than 3.5 g/day after complete or partial remission.

### 2.4. Statistical analysis

All statistical analyses were performed using Stata software (ver. 14.0; StataCorp, College Station, TX, USA). Descriptive statistics are provided as mean and standard deviation (SD) or median and interquartile range (IQR) or range. We pooled the ratios for the clinical response parameters. The statistical heterogeneity of the included studies was measured using the chi-squared-based Q-test and classified based on the I^2^ statistic, as follows: (1) no heterogeneity, I^2^ = 0%–25%; (2) moderate heterogeneity, I^2^ = 25%–50%; (3) high heterogeneity, I^2^ = 50%–75%; and (4) extreme heterogeneity, I^2^ = 75%–100%. We used a random-effects model for data analysis when high or extreme heterogeneity was observed (p < 0.1 or I^2^ > 50%). For no or moderate heterogeneity (p > 0.1 or I^2^ < 50%), a fixed-effect model was used.

## 3. Results

### 3.1. Search results

**Figure 1:**
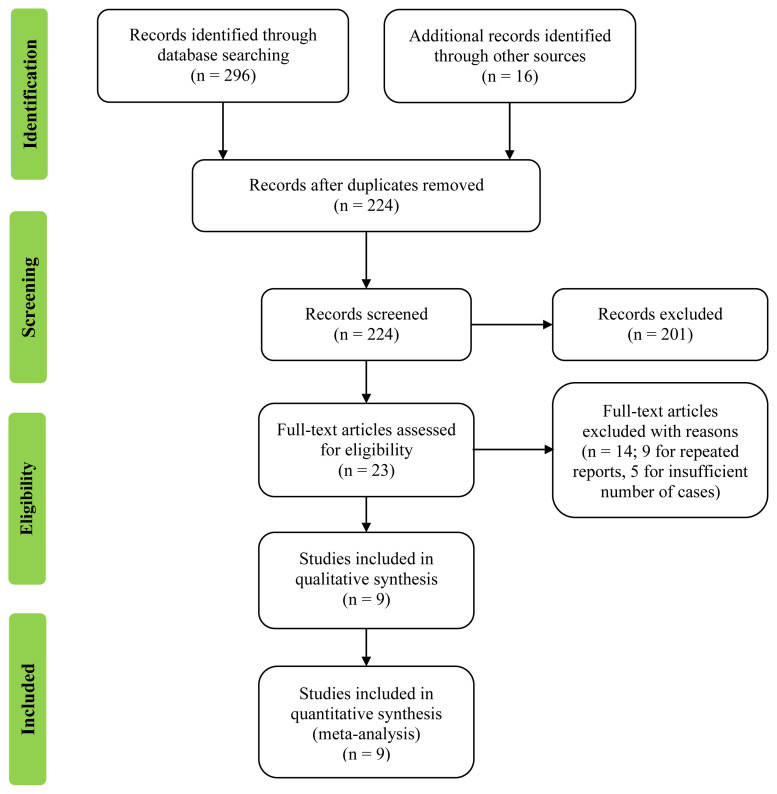
Flow diagram for study selection.

Our electronic database searches and manual screening yielded 312 citations: 72 were excluded as duplicate records and 201 were excluded due to not satisfying the inclusion or exclusion criteria; 23 potentially relevant citations were retrieved as full-text documents and checked in more detail (Figure 1). Fourteen of the full-text documents were excluded: five due to insufficient patient numbers and nine because they were repeat reports. Ultimately, a total of nine studies with 357 patients met the predefined selection criteria (Table).

### 3.2. Characteristics of the included studies

All studies reported the outcomes of IMN patients with NS treated with RTX; there was one matched cohort study, four prospective studies, two RCTs, and two retrospective studies. In the matched-cohort study, IMN patients who received second-line RTX for NS that persisted or relapsed after previous treatment with IST were compared with patients given first-line RTX therapy [12]. In one RCT (GEMRITUX) including IMN patients with persistent NS, the efficacy of a standard dose of RTX provided as two infusions, in addition to supportive therapy, was compared with that of supportive therapy alone [13]. Another RCT (MENTOR) compared the efficacy and safety of RTX with cyclosporine for patients with apparent IMN [14]. One prospective study compared two RTX protocols used to treat patients with IMN [15]. Another retrospective study evaluated the efficacy and safety of RTX for treatment of IMN patients who were nonresponsive to prior IST [16]. The remaining four studies evaluated the same or different RTX protocols for patients with IMN [17–20]. All studies were published between 2008 and 2019; three were multicenter studies [13,14,19] and the others were single-center studies [12,15–18,20]. The sample size ranged from 15 to 100 patients. Three studies reported the outcomes of RTX as treatment for IMN patients who had not received prior IST (first-line RTX therapy) [13,15,18]; one study evaluated the efficacy of RTX as a rescue therapy for IMN patients with a NS that persisted or relapsed after different ISTs (second-line therapy) [16]; and the remaining five studies investigated the response to RTX as a first- and/or second-line therapy [12,14,17,19,20]. All studies included adult patients, with median or mean ages ranging from 47 to 63 years. The baseline median or mean proteinuria range was 8.9 to 13.0 g/d in seven studies and 5.9 to 7.7 g/g of creatinine in two studies [13,15]. The RTX protocols in these studies were classified as follows: (1) low-dose RTX (two studies, one or two 375 mg/m^2^ doses per week [13,19]; (2) standard-dose RTX (four studies, four 375 mg/m^2^ doses per week or treatment based on B-cells [12,16,18,20]; and (3) high-dose RTX (three studies, two 1 g infusions at 2-week intervals [14,15,17]. The follow-up time was 12 or 24 months in five studies [12,14,17–19], while in two 6-month trials it was 15 and 17 months, respectively [13,15]; in two other studies, it was 12 and 29 months (median), respectively [14,16]. The four studies for which quality assessment could be performed [12–15] were rated as high quality, with a mean overall NOS score of 8.75 (IQR: 8.25–9).

### 3.3, Primary outcomes

**Figure 2:**
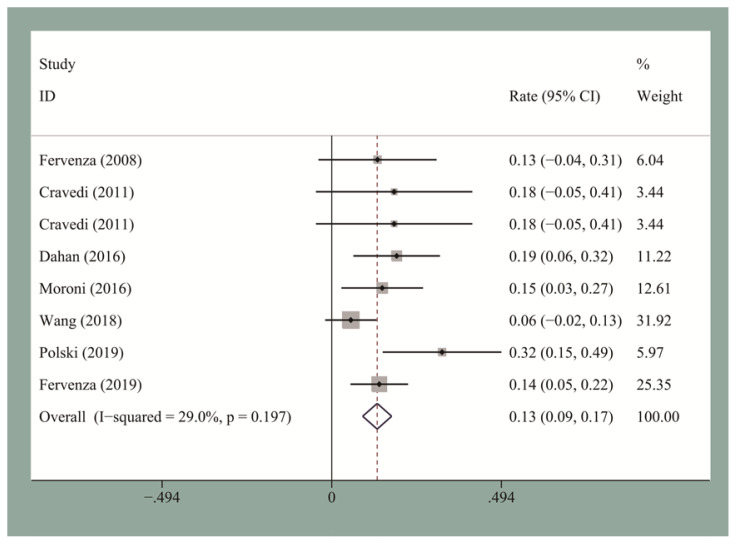
The pooled 12-month CR rate for IMN patients who received RTX treatment.

**Figure 3:**
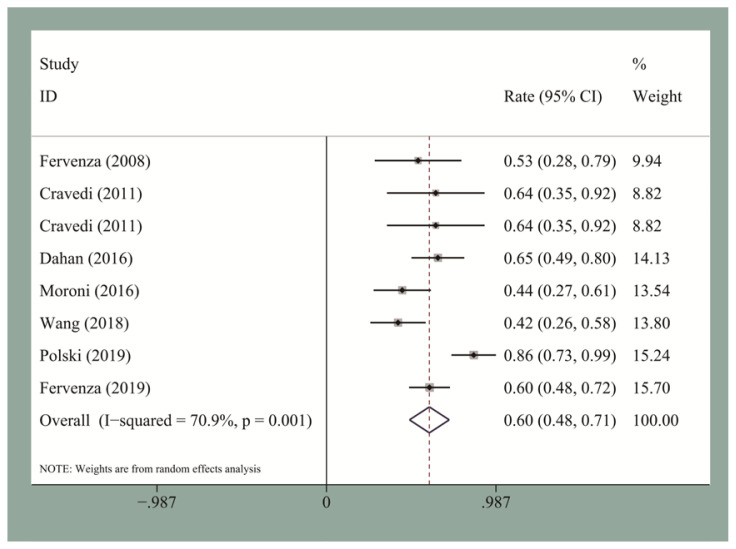
The pooled 12-month OR rate for IMN patients who received RTX treatment.

**Figure 4:**
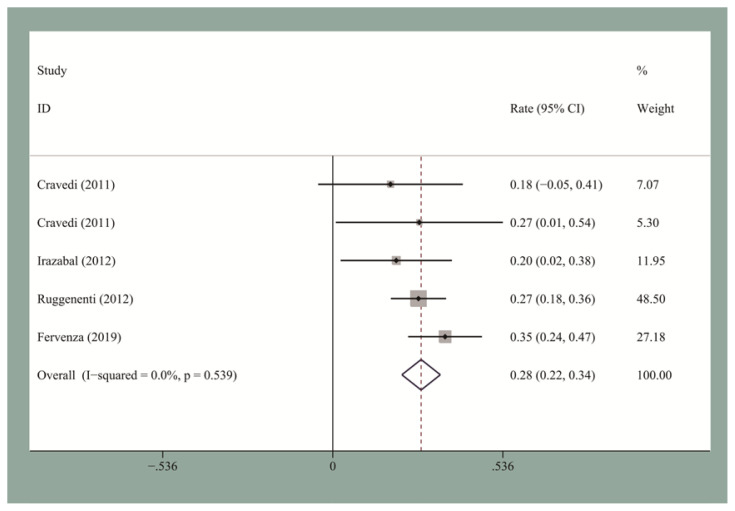
The pooled 24-month CR rate for IMN patients who received RTX treatment.

**Figure 5:**
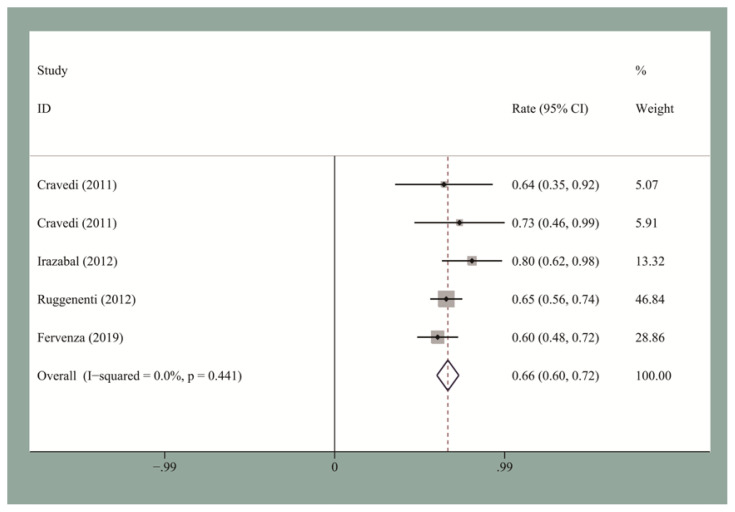
The pooled 24-month OR rate for IMN patients who received RTX treatment.

**Figure 6:**
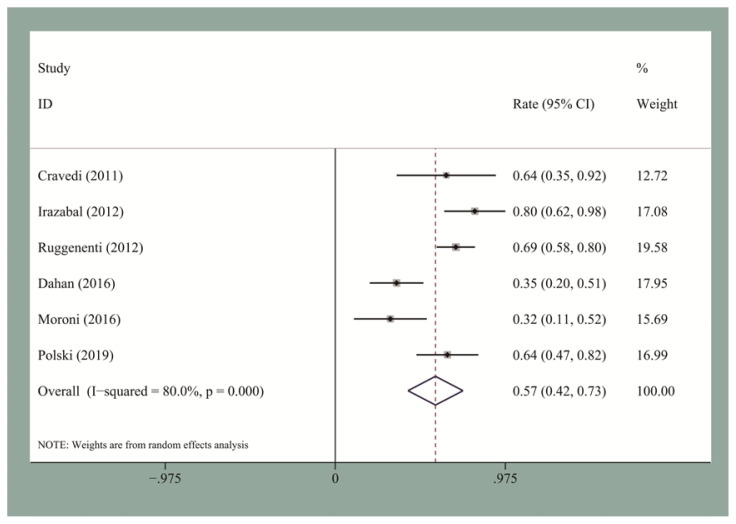
The pooled OR rate for IMN patients who received first-line RTX treatment.

**Figure 7:**
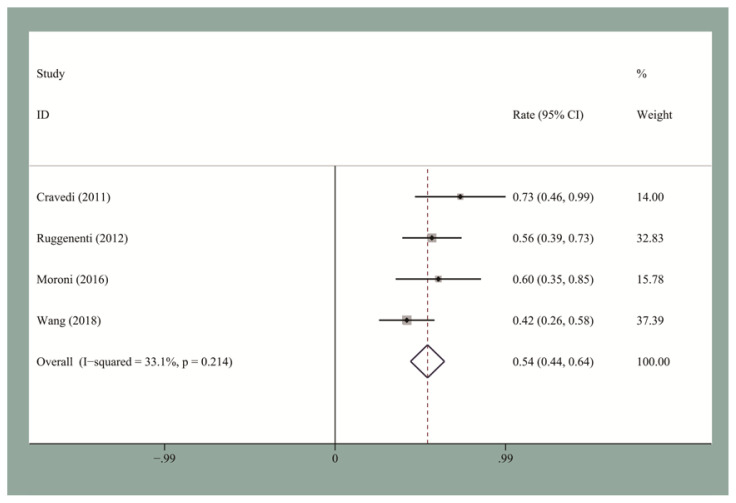
The pooled OR rate for IMN patients who received second-line RTX treatment.

The pooled CR and OR rates at the end of follow-up were 19.5% [95% confidence interval (CI), 0.12–0.27] and 58% (95% CI, 0.53–0.63), respectively. We then performed the publication bias test. Egger’s test for small-study for CR and OR did not suggest a publication bias. The pooled CR and OR rates at 12 months for the seven studies reporting these data were 13.2% (95% CI, 0.09–0.18) and 60% (95% CI, 0.48–0.72), respectively (Figures 2 and 3). The pooled CR and OR rates at 24 months for the four studies reporting these data were 27.8% (95% CI, 0.22–0.34) and 66% (95% CI, 0.6–0.72), respectively (Figures 4 and 5). Subgroup analyses showed that the pooled OR rates for the low-, standard-, and high-dose groups were 39.3% (95% CI, 0.28–0.51), 64% (95% CI, 0.51–0.77), and 60% (95% CI, 0.51–0.7), respectively. The pooled OR rates for the first-line and second-line groups were 58% (95% CI, 0.42–0.73) and 54% (95% CI, 0.44–0.64), respectively (Figures 6 and 7). The median time to remission among the four studies that reported it was 5.5 months (IQR: 3.3–7.1). The median relapse rate among the six studies that reported it was 13.3% (IQR: 6.1–14%), with one study reporting a median relapse time of 42 months (range: 7–116 months).

### 3.4. Secondary outcomes

Six studies reported stabilization or improvement of overall renal function. Six studies also reported significantly increased serum albumin, along with a reduction of proteinuria. Serum cholesterol or triglyceride also decreased in three studies. A reduction of the CD19+ or CD20+ B-cell count was reported in seven studies; the cells were fully or mostly cleared from circulation immediately after the first administration of RTX, and recovered towards normal ranges over 3–9 months. Two studies reported significant increases and decreases, respectively, in serum IgG and IgM levels during treatment, while serum IgA levels remained relatively stable. Three studies also reported PLA2R depletion (median rate, 78%; range: 50–93%).

### 3.5. Safety

Seven studies reported adverse events, most of which were transfusion-related. Of the nonserious adverse events, most were rapidly and completely resolved by reducing the RTX infusion rate or providing supportive treatment. Two studies reported one case each of SAEs (both 3% of all cases). The incidence of SAEs was 17% in one RCT.

## 4. Discussion

Membranous nephropathy (MN) accounts for about 25% of adult cases of NS and is the leading glomerulopathy after kidney transplantation [1]. Recently, great progress has made toward understanding such conditions. The discovery of autoantibodies against PLA2R and THSD7A in serum (and recognition of their contribution to the deposition of immune complexes on the glomerular basement membrane) was a major breakthrough that enhanced our understanding of IMN [3,4]. The presence of those antibodies provides a clear rationale for the use of anti-B-cell therapy. RTX is a human-murine chimeric glycosylated immunoglobulin composed of murine light- and heavy-chain variable region sequences and human kappa and human IgG1 constant region sequences [9]. CD20 is a B-lymphocyte transmembrane protein that is expressed in normal B-cells but not in stem cells, pro-B cells, plasma B cells or other normal tissue cells. Specific affinity of RTX with CD20 on normal B cells elicits circulating and tissue-resident CD20+ cell lysis, but not the destruction of stem cells or normal tissue cells. Depletion of B cells decreases antibody and cytokine production, and affects the process of antigen presentation [9]. Selective depletion of B cells indicates that RTX is a reasonably safe treatment for IMN. Nonprospective studies have reported efficacy of RTX for IMN patients considered for treatment with ISTs, with a remission rate of 60% [20]. RTX seems to be as effective as other immunosuppressive regimens for IMN. However, given the absence of a control group and lack of RCTs, it is possible that the beneficial effect observed may be due to spontaneous remission rather than any therapeutic action of RTX. Furthermore, the optimal dose of RTX for IMN remains unknown because different dosing protocols have been used, ranging from one single dose of 1 g to 4 weekly doses of 375 mg/m^2^. Thus, we conducted this meta-analysis to comprehensively investigate the efficacy and safety of RTX in patients with IMN.

Our pooled OR rates at 12 and 24 months were 60% and 66%, respectively. Although no study has directly compared RTX with alkylating agents, our results were similar to or better than those of the RCTs forming the basis of the KDIGO 2012 recommendation that alkylating agents with cyclophosphamide and steroids be used as the first-line therapy for IMN [6]. However, some issues must be considered when interpreting these results. First, slightly different definitions of remission were used by the included studies, which may complicate the interpretation of the data. Furthermore, two studies lacked long-term follow-up data, with a therapy duration for the test drug of only 6 months. The onset of complete remission induced by RTX may have a lag time of at least 6 months [21]. RTX decreases the number of B cells, and results in a progressive reduction in titers of circulating antibodies and those that are deposited in the subepithelial space. Even if subepithelial antibody deposition stops immediately, the deposits that were already formed are long-lived, such that a slow and progressive decrease in deposits and proteinuria can be expected [22]. Therefore, a 6-month follow-up is unrealistic for gauging therapeutic success, which is supported by the fact that none of the patients in the MENTOR study showed complete remission at 6 months [14]. Fortunately, those studies had a median observational follow-up of > 12 months.

Our subgroup analyses yielded pooled OR rates for the low-, standard-, and high-dose groups of 39.3%, 64%, and 60%, respectively. Low-dose RTX (one or two 375 mg/m^2^ doses per week) was far less effective than the standard- and high-dose regimens. Thus, for the treatment of IMN, the key questions are whether repeated initial dosing is necessary, and whether four 375 mg/m^2^ doses per week is superior to two 1 g infusions at 2-week intervals. RTX was originally approved to treat non-Hodgkin’ s lymphoma and, later, rheumatoid arthritis (one 375 mg/m^2^ dose per week for 4 weeks); related, nonprospective studies and RCTs on RTX of IMN patients used modified versions of these approved regimens. However, a barrier to more widespread use is the high cost of RTX compared with cyclophosphamide. In fact, the initial dosing regimens in the GEMRITUX (two 375 mg/m^2^ infusions separated by 1 week, with the potential for a further reinfusion 6 months later) and MENTOR studies (two 1 g infusions at 2-week intervals, potentially repeated after 6 months) resulted in similar CR and OR rates at the 6–24-month follow-ups [13,14]. In the MENTOR and GEMRITUX studies, no statistically significant differences were observed in the CR and OR rates after redosing at 6 months, suggesting no benefit of a repeat-dosing regimen. Furthermore, some studies have evaluated an RTX regimen where a second dose is prescribed based on B-cell depletion and the proteinuria response [10,12]. Most of those studies found that circulating B cells are cleared within 24 h of a single 375 mg/m^2^ RTX dose, calling into question the need for initial repeat dosing. The initial dose may achieve long-term CR, but can be followed by a second dose if B cells are not completely depleted, and in cases of relapse or PR. In addition, some studies have reported that the response rate to RTX in IMN patients is closely associated with the CD19+ and CD20+ B-cell counts, and anti-PLA2R levels [13,15]; however, this topic was outside the scope of this study.

The pooled OR rate for our first- and second-line groups were 58% and 54%, respectively, suggesting that RTX can also achieve persistent remission in patients previously exposed to other immunosuppressants, and in those who failed to respond to treatment with steroids and alkylating agents. Similarly, a previous study of IMN patients treated with RTX found no difference in the antiproteinuric effect between those who had previously received other immunosuppressants and those who were treatment-naive [12]. Furthermore, RTX can effectively reduce proteinuria, and allows discontinuation of CNI treatment, in cyclosporine- or tacrolimus-dependent IMN patients [23]. The mechanisms underlying the response to RTX when previous treatments, such as steroids, alkylating agents, and other immunosuppressants, failed were at least in partly based on their ability to deplete reactive B cells. Given that proteinuria reduction is always preceded by immediate and sustained depletion of circulating B cells [21], the failure of previous unselective ISTs might be explained by incomplete or transient depletion of autoreactive B cells, whereas complete and sustained depletion of pathogenic B-cell clones could account for the response to RTX.

All of the studies included in our meta-analysis reporting the safety profile demonstrated superior outcomes to those for other immunosuppressive drugs used in the treatment of IMN. RTX seems to be safe for, and well-tolerated by, the majority patients. The main adverse events associated with RTX treatment were transfusion-related reactions, most of which occurred during the first RTX administration. Recovery was achieved only on temporary interruption of the infusion, or with administration of corticosteroids. Premedication (acetaminophen or promethazine) before each infusion, with or without corticosteroids, as well as a slow RTX infusion rate, reduced the rate of adverse events. Although an increase in infection risk after RTX was seen when risk factors were present, we found no significant difference in the adverse event or infection rate between patients treated with RTX and those treated with other types of supportive therapy, among studies that included a control group [13]. In addition, SAEs were rare in all studies except the MENTOR study [14]. However, the decreased rate of these events among patients who achieved remission, and increased rate of adverse events in patients with reactive disease, suggests an association with the underlying disease rather than RTX treatment itself [24]. The increased risk of infection or other SAEs in RTX recipients may depend more on patient characteristics, disease status, or the frequently used combined glucocorticoid treatments than on the cumulative RTX dose [24].

## 5. Conclusion

The efficacy of RTX for treatment of IMN is comparable to that of other ISTs. Furthermore, RTX regimens have the advantages of being steroid-free and having low adverse event and relapse rates. Patients who relapsed or were resistant to other IST agents also responded to RTX. Our results provide support for RTX monotherapy as a third option for induction therapy, as well as an option for rescue therapy. RTX-based regimens and other B-cell-targeted therapies may represent the future of IMN therapy.

## Figures and Tables

**Table:** Characteristics and quality assessment of the studies included in the meta-analysis.

First author	Country	Study center	Publication year	Study design	First-/ second-line	Positive rate of PLA2R	Number of patients	Sex (M/F)	Age (year)
Cravedi (1)	Italy	Single-center	2011	Matched cohort study	First-line	NR	11	10/1	48.7 ± 13.9
Cravedi (2)	Italy	Single-center	2011	Matched cohort study	Second-line	NR	11	10/1	50.2 ± 12.3
Polski	France	Single-center	2019	Prospective study	First-line	100	28	21/7	63.0 (51.0–71.0)
Dahan	France	Multicenter	2016	RCT	First-line	73	37	28/9	53.0 (42.0–63.0)
Fervenza	America	Single-center	2008	Prospective study	First-line+ second-line	NR	15	13/2	47.0 ± 8.0
Irazabal	America	Single-center	2012	Prospective study	First-line	NR	20	17/3	49.0 ± 13.0
Moroni	Italy	Multicenter	2016	Prospective study	First-line + second-line	71	34	23/11	52.8 ± 15.2
Ruggenenti	Italy	Single-center	2012	Retrospective study	First-line + second-line	NR	100	72/28	51.5 ± 5.9
Fervenza	America	Multicenter	2019	RCT	First-line + second-line	77	65	47/18	51.9 ± 12.6
Wang	China	Single-center	2018	Retrospective study	Second-line	94	36	30/6	47.3 ± 17.6

**Table:** Characteristics and quality assessment of the studies included in the meta-analysis (continued).

First author	Baseline proteinuria (g/d)	RTX dose	Follow-up time (month)	Selection/ comparability/results	Score
Cravedi (1)	10.9 (6.6–18.6)	Four weekly doses (375 mg/m^2^ each) or B-cell driven treatment	24	****/**/**	8
Cravedi (2)	10.3 (5.8–13.8)	Four weekly doses (375 mg/m^2^ each) or B-cell driven treatment	24	****/**/**	8
Polski	5.9 (4.9–7.6) (g/g of creatinine)	Two infusions of 1 g at 2-week intervals	6	****/**/***	9
Dahan	7.7 (4.6–10.4) (g/g of creatinine)	Two weekly doses (375 mg/m2 each)	6	****/**/***	9
Fervenza	13.0 ± 5.7	Two infusions of 1 g at 2-week intervals	12	NA	NA
Irazabal	11.9 ± 4.9	Four weekly doses (375 mg/m^2^ each)	24	NA	NA
Moroni	11.9 ± 8.2	One or two biweekly doses (375 mg/m2 each)	12	NA	NA
Ruggenenti	9.1 (5.8–12.8)	Four weekly doses (375 mg/m^2^ each)	29 (median)	NA	NA
Fervenza	8.9 (6.8–12.3)	Two infusions of 1 g at 2-week intervals	24	****/**/***	9
Wang	12.3 ± 5.9	Four weekly doses (375 mg/m^2^ each) or B cell-driven treatment	12 (median)	NA	NA

NR, not reported; NA, not available; PLA2R, phospholipase A2 receptor; RTX, rituximab; RCT, randomized controlled trial.

## Data Availability

Data and any supplementary material related to this article can be obtained from the corresponding author on request.
